# Validation of a French-language version of the health education impact Questionnaire (heiQ) among chronic disease patients seen in primary care: a cross-sectional study

**DOI:** 10.1186/s12955-015-0254-0

**Published:** 2015-05-24

**Authors:** Anne Bélanger, Catherine Hudon, Martin Fortin, José Amirall, Tarek Bouhali, Maud-Christine Chouinard

**Affiliations:** Registered nurse and graduate student, Department of health sciences, Université du Québec à Chicoutimi (UQAC), Quebec, Canada; Family physician and professor, Department of family medicine and emergency medicine, Université de Sherbrooke, Quebec, Canada; Adjunct professor, Department of family medicine and emergency medicine, Université de Sherbrooke, Quebec, Canada; Registered nurse and professor, Department of health sciences, Université du Québec à Chicoutimi (UQAC), Québec, Canada

**Keywords:** French-language questionnaire, Health education, Primary care, Chronic disease

## Abstract

**Background:**

The Health Education Impact Questionnaire (heiQ) allows for the evaluation of the effects of education interventions provided to patients with chronic diseases. This study describes the process for the cross-cultural adaptation and validation of the heiQ into French (heiQ-Fv).

**Methods:**

We undertook a systematic translation process followed by a validation study based on the secondary analysis of cross-sectional data from a longitudinal study. Participants in the validation study were adult patients from primary care clinics in Quebec, Canada, with one or more of the following diseases: diabetes, asthma, chronic obstructive pulmonary disease, cardiovascular disease; or one or more risk factors for these diseases. Main outcomes of the study were the French version of the heiQ-Fv and the validation analyses that included internal consistency, test-retest reliability, confirmatory factor analysis (CFA) and concomitant validity.

**Results:**

The validation analysis was conducted on results from 332 participants. Cronbach’s alphas (internal consistency) for seven domains of the heiQ-Fv varied from 0.80 to 0.89; one domain scored 0.69. The test-retest analysis (n = 50) yielded intra-class correlation coefficients from 0.66 to 0.86. The CFA of the eight heiQ domains with the hypothesis of no correlation between the domains yielded a model that did not exhibit acceptable fit values. A model with the hypothesis of all domains correlated exhibited acceptable fit values (scaled chi-square = 1210.15, degrees of freedom = 712, p < 0.001; CFI = 0.98; RMSEA = 0.06; SRMR = 0.065). Results show a moderate correlation (concomitant validity) between five domains of the heiQ-Fv and the Self-Efficacy for Managing Chronic Diseases. We also found a moderate to strong correlation between the Emotional Wellbeing domain of the heiQ and the Kessler Psychological Distress Scale (K6) (r = 0.61; 95 % CI: 0.52 –0.69, p < 0.01).

**Conclusions:**

The heiQ was translated into French using a rigorous translation process; the French-language version showed good psychometric properties. Health professionals and researchers in primary care settings may use the heiQ-FV to evaluate the impact of educational programs on patients with chronic diseases.

**Electronic supplementary material:**

The online version of this article (doi:10.1186/s12955-015-0254-0) contains supplementary material, which is available to authorized users.

## Background

Chronic diseases (CD) are the main cause of death and invalidity in the world. Of the 57 million deaths that occurred globally in 2008, 36 million were due to CD [1]. The increasing number of patients with CD represents a challenge for the health care system [2], that requires an optimization of available health resources [1]. Health education provided by health professionals can play an important role for these patients [3], to help them manage their situation optimally [4] and thus reduce the impact of CD and their health costs [5].

Indicators or effect measures that allow to describe global changes [6] are required to evaluate the effects of education interventions provided to patients with CD. It is also important to evaluate intermediate changes that happen following the intervention, such as an improvement in quality of life and a decrease in the use of services [7]. In 2007, Osborne and colleagues proposed a tool to measure the effects of health education: the health education impact Questionnaire (heiQ) [8]. Many studies used the heiQ to report the results of education and self-management support interventions [9-13]. The heiQ highlights the different effects of health education programs, in particular empowerment, self-management and acceptance of the illness [7]. The English-language version of this questionnaire shows very good psychometric properties [8].

Two papers reported on the translation and validation of the heiQ in German [7] and in Japanese [5] populations. Both research teams performed a complete translation and cultural adaptation of the instrument and verified the reliability and validity of their translated version for its practical application. The validity of the heiQ in the German and Japanese versions was preserved despite translation.

A recent study used four English to French translations of the heiQ to assess the added-value of back-translation and the use of an expert committee to the content and psychometric properties of a translated multidimensional questionnaire [14]. However, the study did not provide a French version of the heiQ for practical application. The aim of this study was to develop a validated French version of the heiQ that could be used as a practical tool to evaluate the impact of educational programs on chronic diseases in primary care.

We performed the transcultural validation of a French-language version of the heiQ, developed through a systematic translation process, followed by the assessment of the psychometric properties of the translated version (internal consistency, test-retest reliability, confirmatory factor analysis, and concomitant validity) in a population of primary care patients.

## Methods

The cross-cultural validation method used was Hébert et al.’s [15] which includes five steps:

1. Selection of a reliable and valid English-language instrument;
2. Translation-back translation by two translators;
3. Committee review to evaluate both versions;
4. Pretest (with approximately 10 participants);
5. Evaluation of the psychometric properties of the instrument.

### Selecting a reliable and valid English-language instrument

In the first step, we chose an English-language tool that demonstrated good psychometric properties. The health education impact Questionnaire (heiQ) was developed in Australia by a research team lead by Richard H Osborne [8]. This questionnaire consists of 40 questions organized into eight scales and aims to evaluate self-management and empowerment of patients with CD in a context of an education intervention [7]. It was subjected to widespread consultation among health experts and patients having one or more CD, through a rigorous process. The heiQ was validated in a study with 598 participants. It showed good internal consistency with Cronbach alphas varying from 0.70 to 0.89 [8]. Studies on the validation of the English-language version of the heiQ confirmed an eight domain factorial structure [8]. The absence of social desirability was reported in another study [16].

Version 3.0 of the heiQ is composed of 40 questions to which participants respond on a four-point Likert scale ranging from 1 (Completely disagree) to 4 (Completely agree). Complete definitions of each domain of the heiQ are available in the original developmental work of the instrument [8].

### Translation back-translation

In the second step, one translator translated the original English-language version into French. A second independent translator translated the French-language version back into English without having seen the original version.

### Committee review

In step three, a bilingual review committee was set up. It was composed of three health researchers (a family physician, a nursing sciences professor and a professor in occupational therapy), one translator, and one student completing a master of science in nursing working in primary care. All members of the committee have regular contact with patients with CD. Each word and item in the questionnaire was discussed among committee members and all versions of the questionnaire (original English-language version, translated French-language version and the version back-translated into English) were compared to validate each item and make adjustments when needed.

### Pretest

A pretest was conducted with a convenience sample of ten patients recruited by the family physicians of a family medicine group in the Saguenay region of Quebec (Canada). Inclusion criteria were the same as those described below in the section ‘Setting and patients’ of the validation study.

A one-hour cognitive interview was done with each participant who read each question out loud and then expressed what he or she thought of the question. During the pretest, most comments were constructive and allowed us to modify certain items. Questions were simple and easy to understand according to participants. Participants did not appreciate questions that were worded negatively. Certain words like “very” were superfluous and made the question too strong according to some. Following these comments, corrections were made. In total, 16 questions were modified.

### Evaluation of the psychometric properties of the heiQ-Fv

#### Setting and patients

The evaluation of the psychometric properties of the heiQ-Fv was based on the secondary analysis of cross-sectional data provided by a longitudinal study called PR1MaC on the adaptation, implementation and evaluation of an intervention aiming to integrate rehabilitation services into primary care for patients with CD [2]. PR1MaC deployed CD-specific professional services in medical clinics aiming to provide health education to patients with chronic diseases or risk factors. This research was conducted in eight primary care clinics. Inclusion criteria for participating patients were: 1) presence of one or more targeted CD (diabetes, asthma, chronic obstructive pulmonary disease, and cardiovascular disease) or one or more risk factors for these diseases; 2) French as first language; 3) aged between 18 and 75 years; 4) have a potential for rehabilitation. Exclusion criteria were: 1) decompensation (CD); or 2) cognitive problem (s). Potential participants were referred by primary healthcare professionals in participating clinics. Following referral, a research assistant contacted each participant by telephone in order to check for admissibility and interest in participating in the study.

#### Data collection

Participants received a series of questionnaires, including the heiQ-Fv, and the consent form by mail. In addition to the heiQ-Fv, patients completed a sociodemographic questionnaire including questions on sex, age, education, family income, marital status and occupation.

To collect information about the presence of CD in patients, we used the Disease Burden Morbidity Assessment (DBMA) by self-report [17, 18] which includes 21 CD. The DBMA provides a count of the number of conditions as well as a multimorbidity index that takes into account patients’ appreciation of the limitations arising from the presence of CD. In this self-report questionnaire, the subject assesses the degree to which each condition limits his or her daily activities on a five-point descriptive scale in which the first level, “not at all”, has a weight of 1, and the fifth level, “a lot”, has a weight of 5; all other conditions are scored zero. The total DBMA score is the sum of the limitation from all conditions.

Self-efficacy for the management of chronic diseases was evaluated with the French-language version of the Self-Efficacy for Managing Chronic Disease 6-Item Scale (SEM-CD) [19-21]. Psychological distress was assessed with the Kessler Psychological Distress Scale (K6) [22, 23].

A subgroup of 50 randomly-selected participants from the PR1MaC project completed the heiQ-Fv questionnaire twice over a two to four-week interval in order to evaluate the test-retest reliability of the instrument. Participants of this subgroup did not receive any intervention during these weeks.

#### Data analysis

The data were analysed using SPSS 20.0. Questionnaires with missing data were kept, but domains of the heiQ-Fv with one or more missing items were rejected. Internal consistency was calculated using Cronbach’s alpha for each domain. To assess stability over time, interclass correlation coefficients were calculated for each domain.

Confirmatory factor analysis (CFA) using LISREL 9.1 was conducted with all domains of the heiQ. The analysis was carried out with robust maximum likelihood (ML) analysis. Evaluation of model accuracy was based on a chi-square test and model fit indices such as the comparative fit index (CFI), the root mean square error of approximation (RMSEA), and the standardized root mean square residual (SRMR). For model fit to be interpreted as “acceptable,” the CFI needed to be above 0.95, the RMSEA below 0.06, and the SRMR below 0.08 [24]. The scale of the Emotional Wellbeing domain in the heiQ is normally reversed. For the construction of the model, the scale of the emotional wellbeing domain was reversed in a way that higher values meant greater overall health-related positive effect, which is the opposite of the scale normally used in the heiQ.

Pearson correlation analyses were used to evaluate the relationship between all domains of the heiQ as well as to measure the concurrent validity of the domains of the heiQ with two other questionnaires: 1) Self-Efficacy for Managing Chronic Disease 6-Item Scale (SEM-CD); and 2) Kessler Psychological Distress Scale (K6).

Ethics approval for this research was obtained from the ethics review boards of the Centre de santé et de services sociaux de Chicoutimi (CSSSC) and the Université du Québec à Chicoutimi (UQAC) in April 2011. All the participants completed and signed an informed consent form.

## Results

Participant characteristics are presented in Table 1. In total, 337 patients were recruited. Five participants were excluded due to missing sociodemographic data; therefore analysis was conducted on the data from 332 participants. Participants presented between one and 14 CD (mean ± SD: 4.9 ± 2.4).

**Table 1:** Sociodemographic characteristics of participants in the validation study

		Total sample (n = 332)	Test-retest subgroup (n = 50)
Age, years: mean (SD)		52.47 (11.6)	55.7 (9.4)
Sex: male/female		172 / 160	23 / 27
Education: n (%)	High school (not completed)	59 (17.9)	2 (4.0)
	High school (completed)	111 (33.3)	23 (46.0)
	College	98 (29.4)	13 (26.0)
	University	64 (19.4)	12 (24.0)
Annual family income in CAD: n (%)	<20,000)	42 (12.6)	2 (4.2)
	20,000-29,999	34 (10.2)	5 (10.4)
	30,000-39,999	43 (12.9)	5 (10.4)
	40,000-49,999	49 (14.8)	8 (16.7)
	≥50,000	164 (49.5)	29 (58.3)
Marital status: n (%)	Married	245 (73.8)	42 (84.0)
	Single	46 (13.9)	5 (10.0)
	Divorced/separated	25 (7.5)	1 (2.0)
	Widowed	16 (4.8)	2 (4.0)
Occupation: n (%)	Work	194 (58.4)	27 (53.1)
	Without work	53 (16.1)	6 (12.2)
	Retired	85 (25.5)	17 (34.7)
Information about CD^2^: mean (SD)	DBMA^1^	10.31 (7.07)	10.9(7.1)
	Number of CD	4.91 (2.39)	5.4 (2.6)

^1^DBMA - Disease burden morbidity assessment

^2^CD–Chronic diseases

Table 2 presents the mean, standard deviation and range of responses for each domain of the heiQ. The internal consistency of each heiQ domain is shown in Table 3. The Cronbach alphas for the domains varied from 0.80 to 0.89, except for one (Self Monitoring and Insight at 0.69). Table 4 presents the results of the test-retest for each domain, with intraclass correlation coefficients (ICC) that ranged from 0.66 to 0.86.

**Table 2:** Descriptive statistics of the scores obtained on the heiQ-Fv by domain

Domain	n^1^	Mean	Standard deviation	Range
Health directed behaviour	320	2.6	0.8	1 - 4
Positive and active engagement in life	326	3.1	0.5	1.2 - 4
Emotional wellbeing	319	2.7	0.6	1 - 4
Self monitoring and insight	319	3.1	0.4	1.8 - 4
Constructive attitudes and approaches	321	3.1	0.5	1.4 - 4
Skill and technique acquisition	317	2.8	0.5	1.3 - 4
Social integration and support	321	3.0	0.5	1 - 4
Health service navigation	330	3.3	0.4	1.8 - 4

^1^Number of persons without missing values per domain ranged from 317 to 330

**Table 3:** Internal consistency of the heiQ-Fv by domain

Domain	n	α^1^
Health Directed Behaviour	320	0.87
Positive and Active Engagement in Life	326	0.86
Emotional Wellbeing	319	0.87
Self Monitoring and Insight	319	0.69
Constructive Attitudes and Approaches	321	0.81
Skill and Technique Acquisition	317	0.81
Social Integration and Support	321	0.87
Health Service Navigation	330	0.80

^1^ Cronbach alpha

**Table 4:** Results of test-retest reliability of the heiQ-Fv by domain

Domain	n	ICC^1^	95 % CI^2^	p value
Health Directed Behaviour	50	0.66	0.41-0.81	<0.01
Positive and Active Engagement in Life	50	0.79	0.64-0.88	<0.01
Emotional Wellbeing	50	0.86	0.76-0.92	<0.01
Self Monitoring and Insight	50	0.79	0.63-0.88	<0.01
Constructive Attitudes and Approaches	50	0.67	0.43-0.82	<0.01
Skill and Technique Acquisition	50	0.84	0.71-0.91	<0.01
Social Integration and Support	50	0.85	0.74-0.92	<0.01
Health Services Navigation	49	0.81	0.67-0.90	<0.01

^1^ ICC = Intraclass correlation coefficient

^2^ CI = Confidence interval

The CFA of the eight heiQ-Fv domains with the hypothesis of no correlation between the domains yielded a model that did not exhibit acceptable fit values (scaled chi-square = 2205, degrees of freedom = 740, p < 0.001; CFI = 0.94; RMSEA = 0.09; SRMR = 0.27). A model with the hypothesis of all domains correlated exhibited acceptable fit values (scaled chi-square = 1210.15, degrees of freedom = 712, p < 0.001; CFI = 0.98; RMSEA = 0.06; SRMR = 0.065). Factor loadings for both models are shown in Table 5. We evaluated the correlations between the dimensions with a correlation matrix produced by the model (Table 6). The lowest correlation observed in the correlation matrix was between Emotional Wellbeing and Health Service Navigation (0.14). The highest correlations observed in the correlation matrix were between Positive and Active Engagement in Life and Constructive Attitudes and Approaches (0.81), and between Skill and Technique Acquisition and Social Integration and Support (0.79). All correlations were statistically significant in the correlation matrix.

**Table 5:** Question numbers by domain and factor loadings of models with and without correlation between domains

Question number	Factor loadings	
	Model with no correlation between domains	Model with correlation between domains
Health Directed behaviour		
19	0.74	0.72
9	0.82	0.80
1	0.80	0.79
13	0.81	0.84
Positive and Active Engagement in Life		
8	0.86	0.84
2	0.76	0.74
5	0.79	0.78
10	0.62	0.63
15	0.66	0.70
Emotional Wellbeing		
21	0.84	0.85
18	0.74	0.75
12	0.77	0.75
7	0.72	0.72
4	0.45	0.44
14	0.82	0.83
Self Monitoring and Insight		
20	0.47	0.49
3	0.49	0.46
6	0.52	0.46
16	0.64	0.66
11	0.53	0.50
17	0.59	0.63
Constructive Attitudes and Approaches		
40	0.56	0.57
27	0.64	0.65
39	0.85	0.79
34	0.74	0.74
36	0.65	0.72
Skill and Technique Acquisition		
26	0.80	0.79
30	0.65	0.66
25	0.74	0.73
23	0.72	0.74
Social Integration and Support		
28	0.74	0.76
37	0.68	0.69
22	0.73	0.75
35	0.80	0.78
31	0.79	0.77
Health Services Navigation		
29	0.74	0.73
24	0.65	0.67
32	0.79	0.77
33	0.49	0.51
38	0.67	0.67

**Table 6:** Correlation matrix produced by the model with the hypothesis of all domains correlated (all t-values are higher than 1.96 and are, therefore, significant)

				Correlation estimate (standard error) t-value				
	Health Directed Behaviour	Positive and Active Engagement in Life	Emotional Wellbeing^1^	Self Monitoring and Insight	Constructive Attitudes and Approaches	Skill and Technique Acquisition	Social Integration and Support	Health Service Navigation
Health Directed Behaviour	1.00							
	(0.12)							
	8.17							
Positive and Active Engagement in Life	0.62	1.00						
	(0.09)	(0.13)						
	7.37	8.02						
Emotional Wellbeing^1^	0.32	0.49	1.00					
	(0.07)	(0.08)	(0.10)					
	4.54	6.07	9.89					
Self Monitoring and Insight	0.67	0.69	0.36	1.00				
	(0.10)	(0.10)	(0.09)	(0.20)				
	6.53	6.66	3.99	5.06				
Constructive Attitudes and Approaches	0.44	0.81	0.58	0.64	1.00			
	(0.094)	(0.13)	(0.08)	(0.14)	(0.23)			
	4.87	6.40	6.92	4.62	4.29			
Skill and Technique Acquisition	0.55	0.63	0.47	0.87	0.72	1.00		
	(0.09)	(0.09)	(0.07)	(0.14)	(0.13)	(0.14)		
	6.35	6.72	6.36	6.33	5.49	7.22		
Social Integration and Support	0.45	0.71	0.48	0.66	0.74	0.79	1.00	
	(0.08)	(0.09)	(0.08)	(0.12)	(0.12)	(0.11)	(0.14)	
	5.68	7.56	5.95	5.54	6.10	7.52	7.11	
Health Service Navigation	0.25	0.45	0.14	0.53	0.58	0.49	0.56	1.00
	(0.08)	(0.09)	(0.07)	(0.11)	(0.14)	(0.10)	(0.10)	(0.15)
	3.17	4.89	2.08	4.71	4.10	4.94	5.54	6.64

^1^ The scale for Emotional Wellbeing in the heiQ is normally reversed as compared with the other domains. For the construction of the model, the Emotional Wellbeing domain was used with a scale where higher values mean greater overall health-related positive effect, which is the opposite of the scale normally used in the heiQ

Pearson’s correlation coefficients between the scales of the heiQ-Fv are shown in Table 7. In all domains of the heiQ, scales are designed in a way that high values mean positive health-related outcome, except for the domain Emotional Wellbeing, which has a reversed scale where higher values mean greater overall health-related negative effect. This way, positive correlations were observed between all scales, except negative correlations with Emotional Wellbeing. Correlation coefficients ranged from r = 0.09 to r = 0.69. Pearson’s correlation coefficients between the scales of the heiQ-Fv showed the same trend observed in the correlation matrix produced by the model. The lowest correlation was observed between Emotional Wellbeing and Health Service Navigation (r = 0.09). The highest correlations were between Positive and Active Engagement in Life and Constructive Attitudes and Approaches (r = 0.69), and between Skill and Technique Acquisition and Social Integration and Support (r = 0.69).

**Table 7:** Pearson’s correlation coefficients between domains of the heiQ

	Health Directed Behaviour	Positive and Active Engagement in Life	Emotional Wellbeing^1^	Self Monitoring and Insight	Constructive Attitudes and Approaches	Skill and Technique Acquisition	Social Integration and Support	Health Service Navigation
Health Directed Behaviour	1.00	-	-	-	-	-	-	-
Positive and Active Engagement in Life	0.53	1.00	-	-	-	-	-	-
Emotional Wellbeing	0.25	0.40	1.00	-	-	-	-	-
Self Monitoring and Insight	0.51	0.56	−0.20	1.00	-	-	-	-
Constructive Attitudes and Approaches	0.36	0.69	−0.47	0.47	1.00	-	-	-
Skill and Technique Acquisition	0.46	0.57	−0.37	0.62	0.59	1.00	-	-
Social Integration and Support	0.37	0.62	−0.32	0.49	0.63	0.69	1.00	-
Health Service Navigation	0.21	0.37	−0.09	0.43	0.51	0.42	0.47	1.00

^1^ The Emotional Wellbeing domain has a reversed scale where higher values mean greater overall health-related negative effect

Correlation coefficients between all heiQ domains and the Self-Efficacy for Managing Chronic Disease 6-Item Scale (SEM-CD) and the Kessler Psychological Distress Scale (K6) are shown in Table 8. The SEM-CD was moderately correlated with five heiQ domains: Positive and Active Engagement in Life (r = 0.54), Constructive Attitudes and Approaches (r = 0.53), Emotional Wellbeing (r = −0.57), Skill and Technique Acquisition (r = 0.49), and Social Integration and Support (r = 0.46). Among the correlation coefficients observed with the K6, the highest was with the heiQ domain Emotional Wellbeing (r = 0.61).

**Table 8:** Correlation coefficients (r) of the relationship between heiQ domains, the Self-Efficacy for Managing Chronic Disease 6-Item Scale (SEM-CD), and the Kessler Psychological Distress Scale (K6)

Domain	SEM-CD (r)	95 % CI^1^	K6 (r)	95 % CI
Health Directed Behaviour	0.35	0.24 – 0.45	−0.21	−0.32 to −0.09
Positive and Active Engagement in Life	0.54	0.43 – 0.62	−0.46	−0.55 to −0.35
Emotional Wellbeing^2^	−0.57	−0.66 to −0.46	0.61	0.52 – 0.69
Self-Monitoring and Insight	0.37	0.26 – 0.47	−0.20	−0.31 to −0.09
Constructive Attitudes and Approaches	0.53	0.45 – 0.60	−0.45	−0.54 to −0.35
Skill and Technique Acquisition	0.49	0.40 – 0.56	−0.37	−0.46 to −0.28
Social Integration and Support	0.46	0.36 – 0.55	−0.40	−0.50 to −0.30
Health Service Navigation	0.27	0.16 – 0.38	−0.13	−0.24 to −0.01

^1^CI = Confidence interval

^2^The emotional wellbeing domain has a reversed scale where higher values mean greater overall health-related negative effect

## Discussion

The results of this study show that the heiQ-Fv presented good psychometric properties (see the heiQ-Fv final version in Additional file 1). In addition to the initial studies that lead to the development of the heiQ by Osborne and colleagues [8, 25, 26], Schuler et al. and Morita et al. translated and validated the heiQ into German [7] and Japanese [5] respectively. Only the study conducted by Morita [5] presents the values obtained for the different domains of version 3.0 of the heiQ. The mean and standard deviations of the heiQ values obtained in our study are higher than those presented by Morita [5] for all domains. This difference may indicate that our sample of participants was already in a state of empowerment.

In regard to internal consistency, the Cronbach alphas in this study are all above 0.81, with four domains ranking over 0.86. Only one domain is an exception, Self Monitoring and Insight, with a Cronbach alpha at 0.69. This domain was the one in which all questions had rather low factor loadings (from 0.46 to 0.66) in the CFA model. This was also the case for Osborne et al.’s study [8]. Overall, the Cronbach alphas in this study are comparable to those described by Osborne et al. [8] and Morita et al. [5], except for two domains. First, for the Skill and Technique Acquisition domain, the Cronbach alpha value was higher than that obtained by Osborne et al. [8], but comparable to that found by Morita et al. [5]. Second, for the Constructive Attitudes and Approaches domain, the Cronbach alpha value was higher than that obtained by Morita et al. [5], but comparable to that found by Osborne et al. [8]. These results seem to indicate that the reliability of the domains was preserved through the French-language translation process. It is interesting to note that the low value of the Cronbach alpha for Self Monitoring and Insight is comparable to all studies examined.

The test-retest reliability for all domains yielded good results. For two domains, values were lower. The first one is Health Directed Behavior with an ICC of 0.66 and the second one was Constructive Attitudes and Approaches with an ICC of 0.67. The values for the test-retest were inferior to those described by Morita et al. [5] for all domains, but results were comparable or superior to those found by Schuler et al.[7], except for the Constructive Attitudes and Approaches domain. It should be noted that Osborne and colleagues did not report results on this subject [8, 25, 26]. One explanation may be found in the difference in delay between both administrations of the heiQ. In Morita et al.’s study, participants completed the questionnaire on-line two days after having completed it the first time, while in this study, the time span between both completions of the questionnaire varied from 2 to 4 weeks and it was done by mail. Sample size for the test-retest also differed: Morita et al.’s study [5] included 116 participants, while our study included 50. However, according to the method proposed by Hébert et al. [15] this number is sufficient. In the sample of Schuler et al. [7], the time period between the two administrations was three weeks, a timeframe comparable to those used in our study.

In this study, correlation coefficients between domains ranged from r = 0.09 to r = 0.69. The relationship between Positive and Active Engagement in Life and Constructive Attitudes and Approaches was the highest (r = 0.69). For the same relationship, authors of the English version [8] reported a correlation coefficient as high as 0.90 and authors of the German version [7] documented a correlation of 0.85. As discussed by Schuler and colleagues [7], the question arises whether these scales measure different constructs conceptually and empirically. In relation to the correlations observed between domains, the confirmatory factor analysis of the eight heiQ domains with the hypothesis of no correlation between the domains yielded a model that did not exhibit acceptable fit values. However, the model with the hypothesis of all domains correlated exhibited acceptable fit values. These results lead us to the conclusion that the domains in the heiQ-Fv are scales that conceptually and empirically measure constructs which are related to different degrees, which go from a weak relationship (For example: Emotional Wellbeing and Health Service Navigation) to a strong relationship (For example: Positive and Active Engagement in Life and Constructive Attitudes and Approaches).

Concurrent validity of the heiQ-FV domains was assessed with two questionnaires: the SEM-CD-Fv and the K6. The SEM-CD showed a moderate significant relationship with several domains of the heiQ. In the case of the K6 questionnaire, as expected, the highest correlation was observed with the Emotional Wellbeing domain of the heiQ (r = 0.61). The studies by Morita [5] and Schuler [7] compared the domains of their translated versions of the heiQ with several questionnaires, but they were different from those we used in the present study. In Schuler’s study, the heiQ scales showed generally low to moderate correlations with most scales they were compared with. Only one correlation coefficient exceeded 0.60. Comparisons done by Morita and colleagues yielded better results with correlation coefficients ranging from r = 0.40 to r = 0.72.

### Study limits

We followed a rigorous translation and validation method which recommends, if possible, the involvement of the author of the original version of the instrument. Osborne’s team [8] was not involved in the translation process of the heiQ-Fv, but they were aware of our work. Participants in this study were recruited with the requirement that they follow a chronic disease rehabilitation program, which may have increased the motivation of some, and thus created a selection bias in favor of a population with a higher level of empowerment. The fact that this may have led to higher scores in the questionnaire though should not have influenced the validation process. Approximately 60 % of the sample had a mean annual income superior to 50,000 CAD. However, participants were spread across a large age range and a diversity of chronic diseases as usually seen in primary care.

## Conclusion

The French-language version of the heiQ developed and validated among a primary care clientele presenting one or more CD, or their risk factors, shows good psychometric properties. The results obtained allow for the use of this French-language version of the questionnaire, in primary care for patients with CD, to evaluate the impact of health education initiatives.

## Additional file

Additional file 1: Health Education Impact Questionnaire French version (heiQ-Fv).

## Abbreviations

CD: chronic diseases
CI: confidence intervals
CSSSC: Centre de santé et de services sociaux de Chicoutimi
DBMA: Disease Burden Morbidity Assessment
heiQ: health education impact Questionnaire
heiQ-Fv: health education impact Questionnaire – French version
ICC: intraclass correlation coefficients
K6: Kessler Psychological Distress Scale
SEM-CD: Self-Efficacy for Managing Chronic Disease 6-Item Scale
