# Supplementary material for: Validation of a French-language version of the health education impact Questionnaire (heiQ) among chronic disease patients seen in primary care: a cross-sectional study
Source: Health Qual Life Outcomes. 2015 May 24;13:64. doi: 10.1186/s12955-015-0254-0 (PMC4549914; doi:10.1186/s12955-015-0254-0)
Supplement: Additional file 1: — Health Education Impact Questionnaire French version (heiQ-Fv). [file 12955_2015_254_MOESM1_ESM.docx]

Additional file 1. Health Education Impact Questionnaire French version (heiQ-Fv)

| 1 | La plupart des jours de la semaine, je fais au moins une activité pour améliorer ma santé (par exemple : marche, relaxation, exercice) | ⬜ Fortement en désaccord  ⬜ En désaccord  ⬜ En accord  ⬜ Fortement en accord |
| --- | --- | --- |
| 2 | La plupart des jours de la semaine, je fais certaines choses que j’apprécie vraiment | ⬜ Fortement en désaccord  ⬜ En désaccord  ⬜ En accord  ⬜ Fortement en accord |
| 3 | En plus de voir mon médecin, je surveille régulièrement les changements au niveau de ma santé | ⬜ Fortement en désaccord  ⬜ En désaccord  ⬜ En accord  ⬜ Fortement en accord |
| 4 | Je m’inquiète souvent au sujet de ma santé | ⬜ Fortement en désaccord  ⬜ En désaccord  ⬜ En accord  ⬜ Fortement en accord |
| 5 | J’essaie de profiter de ma vie le plus pleinement possible | ⬜ Fortement en désaccord  ⬜ En désaccord  ⬜ En accord  ⬜ Fortement en accord |
| 6 | Je sais ce qui peut déclencher mes problèmes de santé et les aggraver | ⬜ Fortement en désaccord  ⬜ En désaccord  ⬜ En accord  ⬜ Fortement en accord |
| 7 | Mes problèmes de santé me rendent insatisfait de ma vie | ⬜ Fortement en désaccord  ⬜ En désaccord  ⬜ En accord  ⬜ Fortement en accord |
| 8 | Je fais des choses intéressantes dans ma vie | ⬜ Fortement en désaccord  ⬜ En désaccord  ⬜ En accord  ⬜ Fortement en accord |
| 9 | Je fais au moins un type d’activité physique à chaque jour pendant au moins 30 minutes (par exemple : marche, jardinage, tâche domestique, golf, quilles, Tai-chi, natation) | ⬜ Fortement en désaccord  ⬜ En désaccord  ⬜ En accord  ⬜ Fortement en accord |
| 10 | J’ai l’intention de faire des choses agréables dans les prochains jours | ⬜ Fortement en désaccord  ⬜ En désaccord  ⬜ En accord  ⬜ Fortement en accord |
| 11 | Je comprends bien quand et pourquoi je dois prendre ma médication | ⬜ Fortement en désaccord  ⬜ En désaccord  ⬜ En accord  ⬜ Fortement en accord |
| 12 | Je me sens souvent en colère lorsque je pense à ma santé | ⬜ Fortement en désaccord  ⬜ En désaccord  ⬜ En accord  ⬜ Fortement en accord |
| 13 | La plupart des jours de la semaine, je me garde du temps pour des activités saines (par exemple : marche, relaxation, exercice) | ⬜ Fortement en désaccord  ⬜ En désaccord  ⬜ En accord  ⬜ Fortement en accord |
| 14 | Je me sens désespéré (e) en raison de mes problèmes de santé | ⬜ Fortement en désaccord  ⬜ En désaccord  ⬜ En accord  ⬜ Fortement en accord |
| 15 | Je sens que je suis engagée activement dans la vie | ⬜ Fortement en désaccord  ⬜ En désaccord  ⬜ En accord  ⬜ Fortement en accord |
| 16 | Lorsque j’ai des problèmes de santé, je comprends ce que je dois faire pour les contrôler | ⬜ Fortement en désaccord  ⬜ En désaccord  ⬜ En accord  ⬜ Fortement en accord |
| 17 | Je surveille ma santé avec attention et je fais ce qui est nécessaire pour rester en aussi bonne santé que possible | ⬜ Fortement en désaccord  ⬜ En désaccord  ⬜ En accord  ⬜ Fortement en accord |
| 18 | Je suis contrarié (e) quand je pense à ma santé | ⬜ Fortement en désaccord  ⬜ En désaccord  ⬜ En accord  ⬜ Fortement en accord |
| 19 | Je fais de la marche comme exercice, pendant au moins 15 minutes par jour, la plupart des jours de la semaine | ⬜ Fortement en désaccord  ⬜ En désaccord  ⬜ En accord  ⬜ Fortement en accord |
| 20 | Considérant ma santé, j’ai des attentes réalistes par rapport à ce que je peux et ne peux pas faire | ⬜ Fortement en désaccord  ⬜ En désaccord  ⬜ En accord  ⬜ Fortement en accord |
| 21 | Si je pense à ma santé, je me sens déprimé (e) | ⬜ Fortement en désaccord  ⬜ En désaccord  ⬜ En accord  ⬜ Fortement en accord |
| 22 | Si j’ai besoin d’aide, j’ai des gens sur qui je peux compter | ⬜ Fortement en désaccord  ⬜ En désaccord  ⬜ En accord  ⬜ Fortement en accord |
| 23 | J’ai des moyens efficaces pour que mes symptômes (par exemple : inconfort, douleur et stress) ne limitent pas ce que je peux faire dans ma vie | ⬜ Fortement en désaccord  ⬜ En désaccord  ⬜ En accord  ⬜ Fortement en accord |
| 24 | J’ai des relations positives avec mes professionnels de la santé | ⬜ Fortement en désaccord  ⬜ En désaccord  ⬜ En accord  ⬜ Fortement en accord |
| 25 | J’ai une bonne idée de comment gérer mes problèmes de santé | ⬜ Fortement en désaccord  ⬜ En désaccord  ⬜ En accord  ⬜ Fortement en accord |
| 26 | Lorsque j’ai des symptômes, j’ai les habilités pour m’aider à y faire face | ⬜ Fortement en désaccord  ⬜ En désaccord  ⬜ En accord  ⬜ Fortement en accord |
| 27 | J’essaie de ne pas laisser mes problèmes de santé m’empêcher de jouir de la vie | ⬜ Fortement en désaccord  ⬜ En désaccord  ⬜ En accord  ⬜ Fortement en accord |
| 28 | J’ai suffisamment d’amis pour m’aider à faire face à mes problèmes de santé | ⬜ Fortement en désaccord  ⬜ En désaccord  ⬜ En accord  ⬜ Fortement en accord |
| 29 | Je communique de façon confiante avec mon médecin au sujet de mes besoins de santé | ⬜ Fortement en désaccord  ⬜ En désaccord  ⬜ En accord  ⬜ Fortement en accord |
| 30 | J’ai une bonne compréhension des équipements qui pourrait me faciliter la vie | ⬜ Fortement en désaccord  ⬜ En désaccord  ⬜ En accord  ⬜ Fortement en accord |
| 31 | Lorsque je me sens malade, ma famille et les personnes qui prennent soin de moi comprennent ce que je vis | ⬜ Fortement en désaccord  ⬜ En désaccord  ⬜ En accord  ⬜ Fortement en accord |
| 32 | Je donne avec confiance aux professionnels de la santé l’information dont ils ont besoins pour m’aider | ⬜ Fortement en désaccord  ⬜ En désaccord  ⬜ En accord  ⬜ Fortement en accord |
| 33 | Mes besoins sont satisfaits par les ressources en santé disponibles (par ex: médecins, hôpitaux et services communautaires) | ⬜ Fortement en désaccord  ⬜ En désaccord  ⬜ En accord  ⬜ Fortement en accord |
| 34 | Mes problèmes de santé ne gâchent pas ma vie | ⬜ Fortement en désaccord  ⬜ En désaccord  ⬜ En accord  ⬜ Fortement en accord |
| 35 | En général, je sens que mes amis et ma famille veillent bien sur moi | ⬜ Fortement en désaccord  ⬜ En désaccord  ⬜ En accord  ⬜ Fortement en accord |
| 36 | Je trouve que j’ai une belle vie même quand j’ai des problèmes de santé | ⬜ Fortement en désaccord  ⬜ En désaccord  ⬜ En accord  ⬜ Fortement en accord |
| 37 | J’ai assez d’occasions de parler de mes problèmes de santé avec des gens qui me comprennent | ⬜ Fortement en désaccord  ⬜ En désaccord  ⬜ En accord  ⬜ Fortement en accord |
| 38 | Je collabore avec mes médecins et autres professionnels de la santé | ⬜ Fortement en désaccord  ⬜ En désaccord  ⬜ En accord  ⬜ Fortement en accord |
| 39 | Je ne laisse pas mes problèmes de santé contrôler ma vie | ⬜ Fortement en désaccord  ⬜ En désaccord  ⬜ En accord  ⬜ Fortement en accord |
| 40 | Si d’autres peuvent faire face à des problèmes comme les miens, je le peux aussi | ⬜ Fortement en désaccord  ⬜ En désaccord  ⬜ En accord  ⬜ Fortement en accord |
